# Decomposing the Mechanism of Qishen Granules in the Treatment of Heart Failure by a Quantitative Pathway Analysis Method

**DOI:** 10.3390/molecules23071829

**Published:** 2018-07-23

**Authors:** Weiquan Ren, Sheng Gao, Huimin Zhang, Yinglu Ren, Xue Yu, Weili Lin, Shuzhen Guo, Ruixin Zhu, Wei Wang

**Affiliations:** 1 School of Traditional Chinese Medicine, Beijing University of Chinese Medicine, Beijing 100029, China; rwq0931@bucm.edu.cn (W.R.); hmzhang@bucm.edu.cn (H.Z.); renyl@bucm.edu.cn (Y.R.); loving0506@126.com (X.Y.); 2 School of Life Sciences and Technology, Tongji University, Shanghai 200092, China; 1453020@tongji.edu.cn; 3 Key Laboratory of Computational Biology, CAS-MPG Partner Institute for Computational Biology, Shanghai Institutes for Biological Sciences, Chinese Academy of Sciences, Shanghai 200032, China; linweili_tj@163.com; 4 School of Life Sciences, University of Chinese Academy of Sciences, Beijing 100049, China

**Keywords:** Qishen granules, heart failure, quantitative pathway analysis, decomposing formula, Wenyang Yiqi Huoxue, Qingre Jiedu

## Abstract

Qishen granules (QSG) have beneficial therapeutic effects for heart failure, but the effects of decomposed recipes, including Wenyang Yiqi Huoxue (WYH) and Qingre Jiedu (QJ), are not clear. In this study, the efficacy of WYH and QJ on heart failure is evaluated by using transverse aortic constriction (TAC) induced mice and the significantly changed genes in heart tissues were screened with a DNA array. Furthermore, a new quantitative pathway analysis tool is developed to evaluate the differences of pathways in different groups and to identify the pharmacological contributions of the decomposed recipes. Finally, the related genes in the significantly changed pathways are verified by a real-time polymerase chain reaction and a Western blot. Our data show that both QJ and WYH improve the left ventricular ejection fraction, which explain their contributions to protect against heart failure. In the energy metabolism, QJ achieves the therapeutic effects of QSG through nicotinamide nucleotide transhydrogenase (Nnt)-mediated mechanisms. In ventricular remodeling and inflammation reactions, QJ and WYH undertake the therapeutic effects through 5′-nucleotidase ecto (Nt5e)-mediated mechanisms. Together, QJ and WYH constitute the therapeutic effects of QSG and play important roles in myocardial energy metabolism and inflammation, which can exert therapeutic effects for heart failure.

## 1. Introduction

As an incurable and progressive disease, heart failure (HF) has high morbidity and mortality rates in most countries including China [1]. According to previous studies, the prevalence of HF ranges from 1% to 2% and increases among people of age 65 years and older [2]. Chronic HF has a similar prevalence, of 1% to 2% and, among such cases, approximately 50% of cases involve heart failure with a reduced ejection fraction (HFrEF) [3]. The onset of the disease is indicated by subclinical myocardial hypertrophy, the activation of intracellular signaling pathways, and the release of neurohumoral factors; however, left ventricular (LV) systolic function is still preserved [4].

Myocardial hypertrophy plays an important role in heart failure and is an abnormal enlargement of the heart muscle in response to a chronic increase in cardiac workload, which is commonly caused by physiological conditions, such as the amount of exercise performed by an athlete, or pathological disturbances, such as complications of hypertension, valvular heart disease, and other cardiovascular diseases [5]. Ventricular remodeling-induced myocardial hypertrophy is a complex pathophysiologic process characterized by changes in the cellular (tissue) and molecular levels. These changes result in cardiac dilatation and the deterioration of left ventricle (LV) function and they contribute to the development of heart failure [6]. The development of HF is related to a number of signaling cascades including inflammation, energy metabolism, and more. However, relatively little is known about the regulation of pathways and molecular events as well as the time-dependent activation during the onset of heart failure and the progression of HF development [7,8,9]. Furthermore, strategies that prevent and reverse myocardial hypertrophy have attracted attention due to the rising cost of this health burden [10].

Multi-target treatments could be more effective in HF, since it is related to multiple risk factors [11]. Traditional Chinese medicine played an important role in treating heart failure, which has been proven in both the clinical trial space and in practice. According to the differentiation of syndromes, various treatment principles and related herbs were used in different trials. Among them, warming Yang, nourishing Qi, and promoting blood circulation to remove stasis (Wenyang Yiqi Huoxue, WYH) were widely used to treat heart failure in China for many years. Today, heat-clearing and detoxification (Qingre Jiedu, QJ) are considered a promising new method for heart failure and have gained more attention [12]. Qishen granules (QSG) are composed of *Radix Astragali Mongolici*, *Salvia miltiorrhiza bunge*, *Flos Lonicerae,* and other herbs. These granules are used for warming Yang, nourishing Qi, promoting blood circulation to remove stasis, heat-clearing, and detoxification. As shown in our previous studies, some active compounds of QSG including luteolin, cryptotanshinone, licochalcone A, glycyrrhetinic acid, salsolinol, isoacid chlorogenic C, salvianolic acid A, and salvianolic acid B, which all have been validated by direct biochemical methods [13,14]. Furthermore, QSG prevents the development of myocardial hypertrophy and fibrosis, by multiple pharmacological mechanisms, in the midst of heart failure induced by chronic myocardial ischemia, myocardial infarction, and an excessive afterload [15,16,17,18,19,20,21]. There have been some studies that explored the effects of QSG using heart failure models of mice or rats [22,23]. They found the efficacy of QSG for different features such as inhibiting inflammation injury and restoring Ca^2+^ uptake. However, the effects of decomposed recipes of QSG including WYH and QJ are not clear. The principle of Chinese formula compatibility was efficacy-oriented [24]. The recipes of QSG undertake their own therapeutic effects and contribute to the pharmacological mechanism of the formula. By decomposing QSG into QJ and WYH, we looked to explore the molecular mechanism of the decomposed recipes in the treatment of heart failure.

In the current study, the highly reproducible mice model of transverse aortic constriction (TAC) is used to mimic left ventricular hypertrophy and heart failure [25] and evaluate the effects of QSG, QJ, and WYH. Furthermore, we develop a novel way to quantify the activity of the pathway with gene expression and identify the differences of significantly enriched pathways in the different treatment groups. Finally, we uncover the contributions of QJ and WYH to the effects of QSG on heart failure.

## 2. Results

### 2.1. Echocardiographic Assessment of Left Ventricular Function

Table 1 shows the results of left ventricular function four weeks after surgery in the various treatment groups. Four weeks after surgery, the constriction line was observed at the bifurcation of the arteria anonyma and the left carotid artery was observed in sections of the aortic arch. The aortic blood flow rate at the constriction point was greater than 2400 mm/s, while in the Control group it was less than 900 mm/s. Consequently, this surgery clearly increased the cardiac afterload. At four weeks after surgery, the left ventricular internal diameter end systole (LVIDs) and diastole (LVIDd), the left ventricular posterior wall thickness end systole (LVPWs) and diastole (LVPWd), the left ventricular anterior wall thickness end systole (LVAWs) and diastole (LVAWd), the left ventricular end-systolic volume (LVESV) and -diastolic volume (LVEDV), and the left ventricular mass (LVM) were all significantly increased in the Model group when compared with the values in the Control group (*p* < 0.01). The ejection fraction (EF) and left ventricular fraction shortening (FS) significantly decreased (*p* < 0.01). The LVIDs and LVESV decreased in the QSG group four weeks after surgery compared with those of the Model group (*p* < 0.05). Moreover, the LVPWs, LVPWd, LVAWd, LVAWs, and LVM significantly decreased (*p* < 0.01). The EF and FS increased (*p* < 0.05) while the LVEDV and LVIDd were not significantly different. The LVIDd, LVPWs, and LVEDV decreased in the WYH group four weeks after surgery when compared with those of the Model group (*p* < 0.05). Furthermore, the LVIDs, LVPWd, LVAWd, LVAWs, LVESV, and LVM decreased significantly (*p* < 0.01). The EF and FS increased (*p* < 0.01) while the LVIDd, LVAWd, and LVEDV decreased in the QJ group four weeks after surgery. These findings were compared with the values of the Model group (*p* < 0.05). Moreover, the LVIDs, LVPWd, LVPWs, LVAWs, LVESV, and LVM decreased significantly (*p* < 0.01). The FS increased (*p* < 0.05) and the EF increased significantly (*p* < 0.01). The LVIDd, LVPWs, LVAWs, and LVEDV decreased in the Fosinopril sodium group four weeks after surgery and compared with those of the Model group (*p* < 0.05). Moreover, the LVIDs, LVPWd, LVAWd, LVESV, and LVM decreased significantly (*p* < 0.01). The EF and FS increased significantly (*p* < 0.01).

For the Model group, the weights of the hearts increased and the ventricular walls were thicker. Additionally, compliance and cardiac function both decreased. However, in the QSG, WYH, QJ, and Fosinopril sodium groups, the granules inhibited the thickening of the left ventricular walls, delayed cardiac remodeling, and improved cardiac function at different levels.

### 2.2. Analysis of Myocardial Hypertrophy

**Figure 1:**
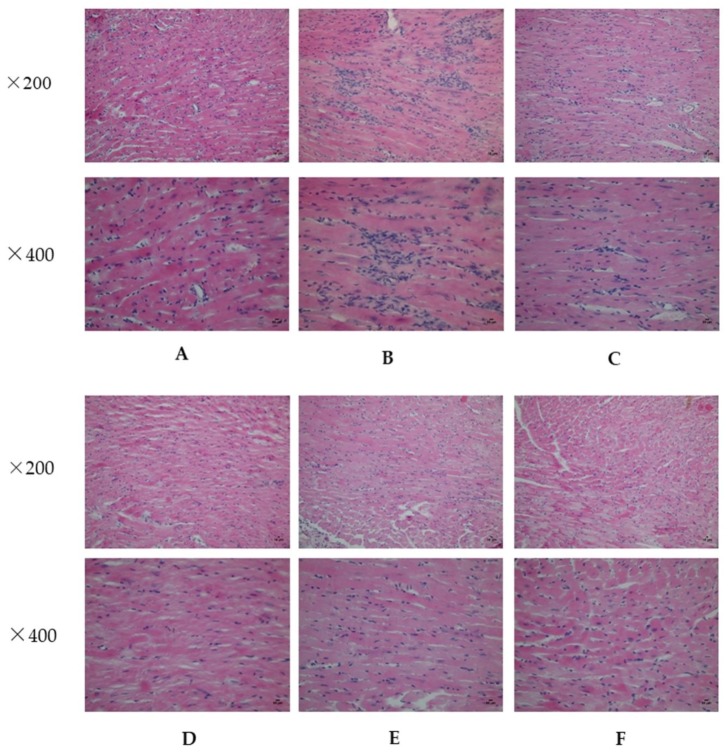
Hematoxylin and eosin staining (HE) of the mouse heart from various treatment groups at the two indicated magnifications (×200 and ×400). (**A**) Sham group; (**B**) Model group; (**C**) Qishen granules (QSG)-treated group; (**D**) Wenyang Yiqi Huoxue (WYH)-treated group; (**E**) Qingre Jiedu (QJ)-treated group; and (**F**) Fosinopril sodium-treated group.

Figure 1 shows the results of hematoxylin and eosin staining (HE), the myocardium of the Sham group (Figure 1A) is normal and myocardial cells are present in an ordered arrangement. The Model group (Figure 1B) shows myocardial cell derangement, hypertrophy, necrosis of a large number of cardiocytes, a loss of normal structure, and muscle fiber dissolution. However, in the treatment groups (Figure 1C–F), the myocardial cells are well arranged and maintain their original forms.

**Figure 2:**
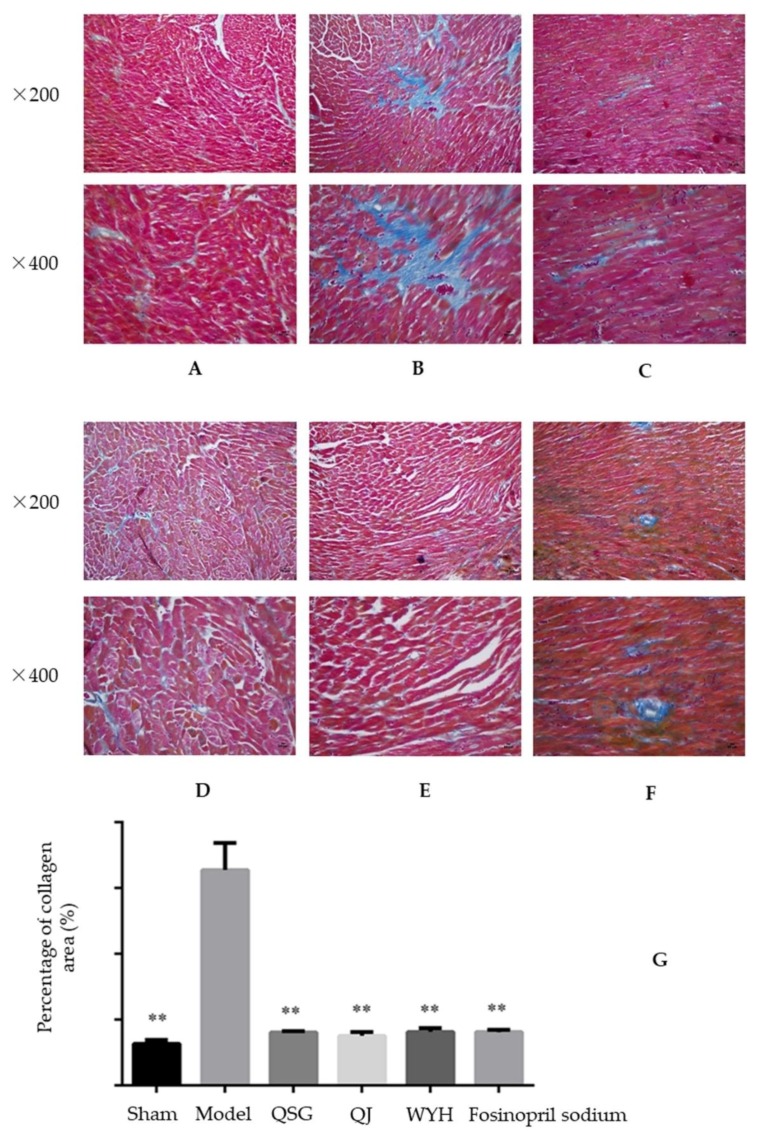
Mallory staining of the mouse heart from various treatment groups at the two indicated magnifications (×200 and ×400). (**A**) Sham group; (**B**) Model group; (**C**) QSG-treated group; (**D**) WYH-treated Group; (**E**) QJ-treated group; and (**F**) Fosinopril sodium-treated group. (**G**) the percentage of collagen in each of these groups. ** *p* < 0.01 relative to the model group. All data are presented as mean ± SE.

Additionally, heart sections were studied with Mallory staining, as shown in Figure 2. In this staining, collagen fibers are blue, nuclei are crimson, and the cytoplasm is red. In the Sham or Control group (Figure 2A), myocardial cells are closely arranged and only a small amount of collagen tissue is distributed in the myocardial interstitium. The Model group (Figure 2B) exhibits significant myocardial hypertrophy and a diffusive growth of collagen fibers. Moreover, the abnormal cells gradually invade and replace the normal tissue. However, in the treatment groups (Figure 2C–F), myocardial cells are closely arranged and only a small amount of collagen tissue is distributed in the myocardial interstitium. Semi-quantitative analysis of the myocardial collagen volume (Figure 2G) shows that, when compared with the Sham group, the collagen area of the Model group is increased significantly (*p* < 0.01). On the other hand, when compared with the Model group, the collagen area of the QSG, QJ, WYH, and Fosinopril sodium groups are significantly reduced (*p* < 0.01).

### 2.3. Transcriptome Results

#### 2.3.1. Differential Expression Analysis

The results of differential expression analysis are shown in the Supplementary Materials, Tables S2–S4. There are 98 differentially expressed genes between the QSG-treatment and Model groups including 44 upregulated genes and 54 downregulated genes. There are 191 differentially expressed genes between the QJ-treatment and Model groups including 49 upregulated genes and 142 downregulated genes. There are 196 differentially expressed genes between the WYH-treatment and Model groups including 49 upregulated genes and 147 downregulated genes. The changes in expression of these genes indicate the impact of the drugs on the disease.

#### 2.3.2. Quantitative Pathway Analysis

A total of 42 pathways are enriched with the differentially expressed genes between the QSG-treatment and Model groups. Forty-five pathways are enriched with the differentially expressed genes between the QJ-treatment and Model groups and 38 pathways are enriched with the differentially expressed genes between the WYH-treatment and Model groups. Pathway enrichment results are shown in Tables S5–S7. Pathway enrichment analysis indicates that the transforming growth factor (TGF)-beta signaling pathway, the regulation of lipolysis in adipocytes, the extracellular matrix (ECM)-receptor interaction, the phosphoinositide 3-kinase (PI3K)-protein kinase B (Akt) signaling pathway, hypertrophic cardiomyopathy (HCM), and the biosynthesis of unsaturated fatty acids related to heart failure were altered. The common disease-related pathways in different treatment groups were compared in a quantitative analysis of the pathways. There are no significant differences in the pathways between the QSG- and QJ-treatment groups as well as between the QSG- and WYH-treatment groups. However, the QJ- and WYH-treatment groups show significant differences in the Foxo signaling pathway, the nicotinate and nicotinamide metabolism pathway, and the ECM-receptor interaction. These pathways are considered to be heart failure-related pathways. The differentially expressed genes in the heart failure-related pathways are shown in Table 2. QJ and WYH may have complementary effects that play roles in myocardial energy metabolism, amino acid metabolism, and the ECM pathway, which are therapeutic for myocardial failure.

### 2.4. Validation of the Key Genes in HF-Related Pathways

**Figure 3:**
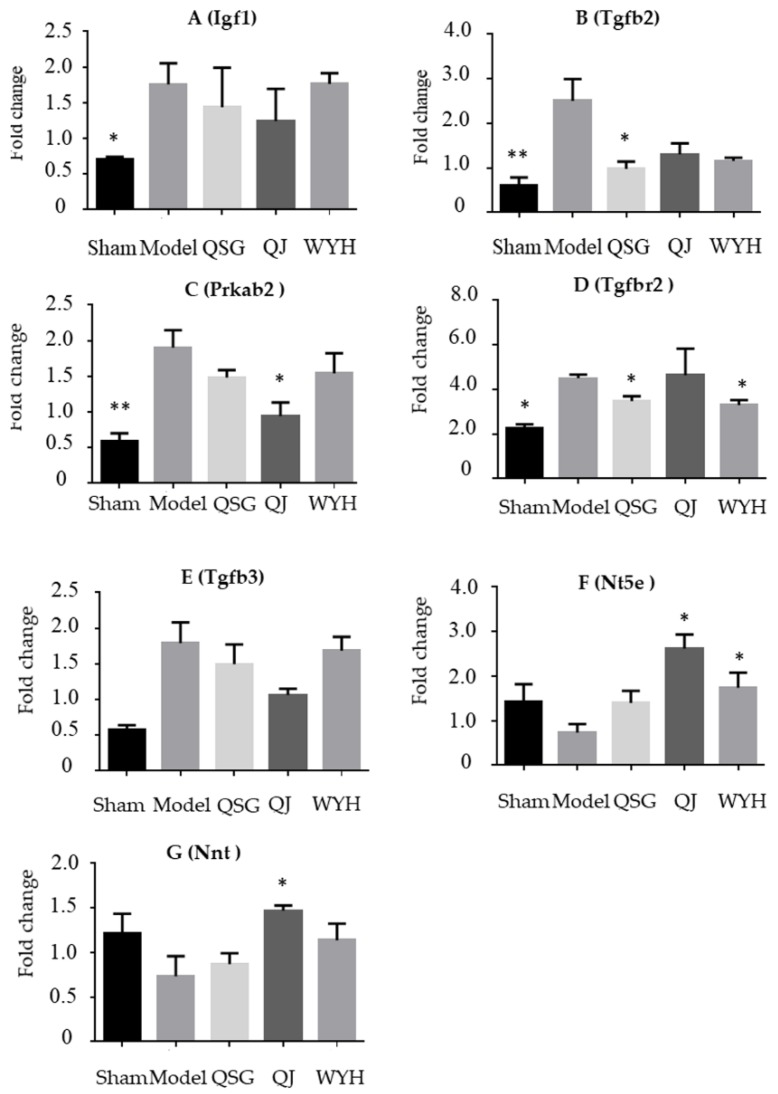
Gene expression in the mouse heart was determined using real-time fluorescent quantitative PCR. Values were generated by converting the estimated marginal means from the RM-ANOVA using a fold change = $2^{-\Delta \Delta \mathrm{CT}}$. The expressions of (**A**) Igf1; (**B**) Tgfb2; (**C**) Prkab2; (**D**) Tgrbr2; (**E**) Tgfb3; (**F**) Nt5e; and (**G**) Nnt genes in the Sham, QSG, QJ, and WYH groups compared with the Model group. * *p <* 0.05 and ** *p* <0.01 relative to the Model group. All data are presented as mean ±SE.

Figure 3 shows gene expression results for the different treatment groups from PCR analysis. Based on PCR results, the expressions of the Igf1 (insulin like growth factor 1) and Tgfbr2 (transforming growth factor beta receptor 2) genes increase (*p* < 0.05), and the expressions of Tgfb2 (transforming growth factor beta 2) and Prkab2 (protein kinase AMP-activated non-catalytic subunit beta 2) genes significantly increase (*p* > 0.01) in the Model group when compared with the Control group. Moreover, the expression of Tgfb3 (transforming growth factor beta 3) is increased and the expression of the Nt5e (5′-nucleotidase ecto) and Nnt (nicotinamide nucleotide transhydrogenase) genes are decreased at insignificant levels. Compared with the expression in the Model group, the expression of the Tgfb2 and Tgfbr2 genes in the QSG group significantly decrease (*p* < 0.05). Additionally, the expression of the Tgfb3 gene and the Igf1 gene decrease, while the expressions of Nt5e and Nnt genes increase. However, these changes are not significant (*p* > 0.05). Compared with expression in the Model group, the expression of the Prkab2 gene significantly decreases and the expression of the Nt5e and Nnt genes increase in the QJ group (*p* < 0.05). The expression of the Igf1, Tgfb2, and Tgfb3 genes decrease at insignificant levels (*p* > 0.05). Compared with expression in the Model group, the expression of the Tgfbr2 gene significantly decrease and expression of the Nt5e gene increases in the WYH group (*p* < 0.05). The expression of Igf1, Tgfb2, Prkab2, and Tgfb3 decrease. The expression of the Nnt gene increases at an insignificant level. The PCR results are consistent with the transcriptome data.

### 2.5. Western Blot Detection of the Nnt and Nt5e Protein Levels

**Figure 4:**
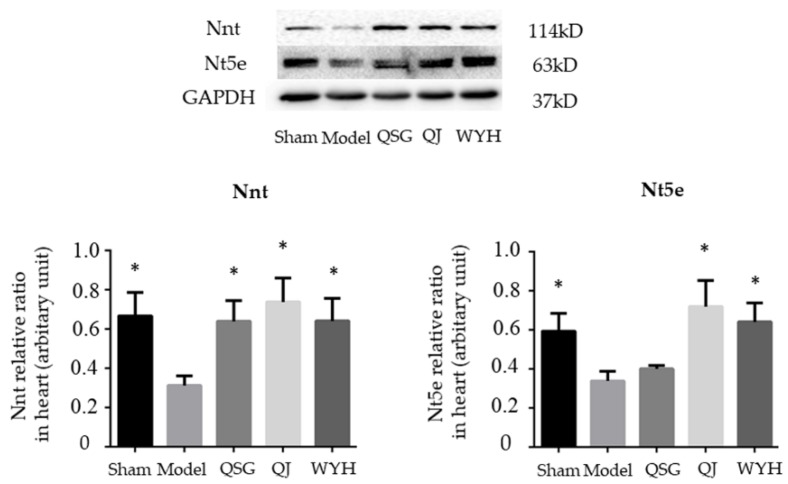
Western blot analysis of the Nnt and Nt5e protein levels in different treated mouse hearts. * *p* < 0.05 relative to the Model group. All data are presented as mean ± SE.

Figure 4 shows results from the Nnt and Nt5e protein levels. The Nnt and Nt5e protein levels significantly decrease in the Model group compared with the levels in the Sham group (*p* < 0.05). Compared with the Model group, the level of Nnt protein significantly increases in the QSG, QJ, and WYH groups and the level of Nt5e protein is significantly increased in the QJ and the WYH groups (*p* < 0.05).

## 3. Discussion

The therapeutic effects of Chinese medicine depend on several components and several targets. Through the study of the decomposition of Chinese medicine by quantitative pathway analysis, the functional pathways of recipes of Chinese medicine can be quantitatively observed and compared. We discovered the molecular mechanisms of the decomposed recipes of QSG, verifying that the complex mechanisms of Chinese medicine can be explained more clearly.

Our main conclusion is that QJ and WYH complementarily contribute to the therapeutic mechanisms of QSG in the treatment of heart failure.

To be specific, the results of the quantitative pathway analysis indicate that QJ and WYH are significantly different in the pathways that affect heart failure. The pathways may play important therapeutic roles in treating heart failure with QJ and WYH. We validate that two key genes, known as Nt5e and Nnt in the nicotinate and nicotinamide metabolism pathway, are affected by the treatments.

In our study, experimental validation shows that Nnt was upregulated significantly in the QJ-treatment groups, which indicates that QJ has a promotion effect on Nnt. Previous studies have shown that the energy consumption rate of mice lacking Nnt decreases, Nnt-PDHC (pyruvate dehydrogenase complex) plays an important role in energy balance, and Nnt can maintain the sustained production of nicotinamide adenine dinucleotide phosphate (NADPH). NADPH produced by Nnt generally contributes to the NADPH pool in the mitochondria and Nnt can be an important source of energy consumption [26,27]. Energy supply plays an important role in heart function; abnormal energy metabolism can be harmful, and the lack of an energy supply may directly affect the heart’s systolic function, which may lead to heart failure [28,29]. Our results show that QJ plays a role in promoting the myocardial energy metabolism by upregulating Nnt, which contributes to therapeutic effects in heart failure.

Inflammation is a compensatory response to injury, but excessive inflammation may damage the heart [30], which contributes to left ventricular hypertrophy, fibrosis, and diastolic dysfunction [31,32]. The rise of inflammatory biomarkers is a significant feature of chronic heart failure. Inflammation has also been proven to cause the development of hypertensive heart failure [33]. Nt5e is upregulated in immune cells and produces extracellular adenosine for treating ventricular remodeling and inflammatory reactions [34,35]. According to our transcriptome data and experimental validation, Nt5e is upregulated in the QJ and WYH groups, which indicates that QJ and WYH have an effect on regulating the cardiac inflammatory response. In addition, the results are verified in our protein experiment.

**Figure 5:**
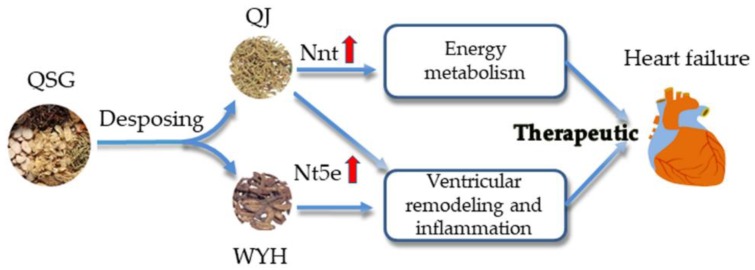
The effects of the decomposed recipes (QJ and WYH) of QSG on heart failure. By studying the decomposed QSG, we found that the two decomposed recipes were therapeutic to heart failure. QJ affects the energy metabolism through Nnt-mediated mechanisms, while QJ and WYH both affect ventricular remodeling and inflammation reactions through Nt5e-mediated mechanisms.

Figure 5 shows a schematic of the main conclusions of our experiments. QJ enhances the energy metabolism efficiency in the treatment of heart failure. Additionally, QJ achieves the therapeutic effects of QSG on the energy metabolism through Nnt-mediated mechanisms. Both QJ and WYH are therapeutic and achieve complementary effects in the treatment of ventricular remodeling and inflammatory reactions. QJ and WYH undertake the effects of QSG on ventricular remodeling and inflammation reactions through Nt5e-mediated mechanisms. Essentially, QJ and WYH together play important roles in QSG that contribute to the therapeutic effect of QSG in treating heart failure.

Due to the various chemical structures of herbs, Chinese formulas and their component recipes usually have multiple effects. Our data also shows the complementary contributions of QJ and WYH to other heart failure-related pathways such as the Foxo signaling pathway and the ECM-receptor interaction. These results may help us find the entire synergic pattern of the different components of QSG. However, further study is still needed.

## 4. Materials and Methods

### 4.1. Animals

All the experiments were conducted in accordance with the “Guiding Principles in the Care and Use of Animals” from the China Physiological Society and received approval from the Animal Care Committee of Beijing University of Chinese Medicine (project identification code: BUCM-4-2015090401-3008, date of approval: 15 September 2015). Sixty male C57BL/6 mice (weighing 16 ± 1 g) were purchased from the Beijing Vital River Laboratory Animal Technology Co. Ltd. (Beijing, China). The animals were housed in clean conditions (certification number SCXK (Jing) 2011-0024) at Beijing University of Chinese Medicine. Conditions were held at a temperature of 22 ± 1 °C, humidity of 55 ± 5%, and under a 12 h light/dark cycle. All mice were allowed free access to tap water and food.

### 4.2. TAC Model Establishment and Grouping

After a one-week acclimation period, the mice were randomly divided into two groups by body weight. The Surgery group held 50 mice while the Sham group held 10 mice. The 50 mice in the Surgery group, in which the TAC model was successful, were further divided into 5 groups including the Model group, the QSG group, the WYH group, the QJ group, and the Fosinopril sodium group. The other 10 mice in the Sham group received similar operations except that they were subjected to stringing without ligation.

Mice were weighed and intraperitoneally injected with pentobarbital sodium (40 mg/kg) to induce anesthesia. Then, these mice were anchored for a thoracotomy and respiration was maintained by a small animal ventilator (SAR-1000 ventilator, CWE, Ardmore, PA, USA). A 5-0 braided silk line (Surgipro, CT, USA) was threaded from the innominate artery and the left common carotid artery bifurcation. It was then was clamped with hook tweezers. Afterward, this string was pulled and wrapped around the aortic arch. The handmade “l” shaped 26-G pad needle was placed over the aortic arch. Both ends of this braided silk thread were clamped and tied on the pad needle. Next, the pad needle was withdrawn, and the extra thread was cut. Lastly, the aortic arch was constricted.

The mice in the Sham group only received a thoracotomy without constriction of the aortic arch, which was followed by chest suturing. The other operations were the same as the Model group. The second day after the surgery, the QSG group was provided with 14.66 g/kg QSG, the WYH group was provided with 14.66 g/kg WYH (a component of QSG), the QJ group was provided with 14.66 g/kg QJ (a component of QSG), and the Fosinopril sodium group was provided with 1.83 mg/kg Fosinopril sodium tablets, which was equal to the clinically effective dose for 4 weeks. The medicine was dissolved in water and administered in a volume of 1.0 mL/100 g. The Control group was provided daily intragastric administration of pure water for 4 weeks, which was the same volume as the Model group. After 4 weeks of oral administration, the mice were subjected to echocardiography and then euthanized. The animals’ chests were quickly opened and the hearts were removed. Afterward, the blood clots in the heart chambers were removed using cold PBS. Next, the connective tissue and blood vessels around the heart were dissected and cleaned. Lastly, the cardiac tissues were weighed and cut horizontally into two parts. The lower part was fixed with paraformaldehyde while the upper part was stored in liquid nitrogen.

### 4.3. Preparation and Determination of the Dose of QSG, QJ, WYH, and Fosinopril Sodium Tablets

The QSG used in the current study was produced by the Beijing University of Chinese Medicine (BUCM, Beijing, China) and was composed of six Chinese herbs including *Radix Astragali Mongolici*, *Salvia miltiorrhiza bunge*, *Flos Lonicerae*, *Scrophularia*, *Radix Aconiti Lateralis Preparata*, and *Radix Glycyrrhizae*. These herbs were purchased from the Beijing Tong Ren Tang Herbal Company (Beijing, China) and verified by qualified experts in the Department of Health and Drugs [13]. WYH consisted of *Radix Astragali Mongolici*, *miltiorrhiza bunge*, and *Radix Aconiti Lateralis Preparata.* The compatibility proportion among these three herbs was decided according to QSG. Similarly, QJ contains *Flos Lonicerae*, *Scrophularia*, and *Radix Glycyrrhizae*. Fosinopril sodium tablets were purchased from Sino American Shanghai Squib Pharmaceutical Ltd. (Shanghai, China).

QSG was prepared by following the previously established procedure. All the prescription herbs were mixed. The herbs were extracted three times with 8 volumes of water and each extraction lasted 1 h. The extracts were then filtered. The aqueous extract was concentrated to a relative density of 1.10–1.15 (60 °C) and then ethanol was added to a 70% final concentration. This was followed by incubation for 16 h to precipitate the extract. After the solution was centrifuged and the precipitate was discarded, the supernatant was concentrated under a reduced pressure and dried in vacuo (60 °C, −0.8 MPa) to obtain a powder of the extract. The product met the quality control criteria established before [15,16]. WYH and QJ were prepared using the same procedure detailed above.

### 4.4. Echocardiographic Assessment of Left Ventricular Function

Echocardiography was conducted four weeks after the operation using a Vevo 2100 ultrasound (Visualsonics, Toronto, ON, Canada) and the corresponding probe (MS-400) with a center frequency of 30 MHz and a standard frame rate of 449 fps. The chest hair was shaved and the mice were anesthetized with isoflurane. The mice were placed in a supine position. We obtained colored Doppler blood flow images of the aortic arch sections left of the ventricular M-mode echocardiography, two-dimensional images of the left parasternal short axis, and the long axis of the left ventricle. In these images, ten cardiac cycles were recorded at every measured point. The left ventricular internal diameter end systole (LVIDs) and diastole (LVIDd), the left ventricular posterior wall thickness end systole (LVPWs) and diastole (LVPWd), and the left ventricular anterior wall thickness end systole (LVAWs) and diastole (LVAWd) were measured. Furthermore, the left ventricular end-systolic volume (LVESV), the left ventricular end-diastolic volume (LVEDV), the left ventricular mass (LVM), the ejection fraction (EF), and the left ventricular fraction shortening (FS) were calculated.

### 4.5. Histopathological Examination

The fixed heart tissues were dehydrated with a gradient ethanol series, embedded in paraffin, sliced, subjected to hematoxylin-eosin (HE) [36] and Mallory staining, and observed under a microscope. The semi-quantitative analysis of myocardial collagen volume fraction, inverted electron microscopy (200-fold), and Mallory staining showed the collagen fibers as blue. Ten non-overlapping fields were randomly selected per slice (every group randomly selected three slices) and the image analysis software Image-Pro plus 6.0 was applied to calculate the percentage of the blue staining area in the entire visual field by performing a semi-quantitative analysis to quantify the volume fraction of cardiac collagen fibers.

### 4.6. Microarray Analysis

#### 4.6.1. RNA Extraction, Amplification, Labeling, and Hybridization

The total RNA was extracted from the frozen cardiac tissue using the TRIzol reagent (Invitrogen, Carlsbad, CA, USA), according to the manufacturer’s instructions. It was digested with DNase I at 37 °C for 15 min to remove any contaminating DNA. The RNA was purified with an RNeasy Kit (Qiagen, Hilden, Germany) and the RNA concentration was measured with a ND-1000 spectrophotometer (NanoDrop Technologies, Wilmington, DE, USA). The RNA quality was evaluated using 1% formaldehyde denaturing gel electrophoresis. The samples used for the microarray analysis had bright ribosomal 28S and 18S RNA bands in a ratio of >1.5:1.

A total of 100 ng of RNA from each sample was used to generate amplified and biotinylated sense-strand cDNAs from the entire expressed genome using the methods described in the GeneChip WT PLUS Reagent Kit User Manual (P/N 703174, Affymetrix Inc., Santa Clara, CA, USA). The Affymetrix Mouse Transcriptome Array 1.0 was hybridized for 16 h in a 45 °C incubator (Affymetrix GeneChip Hybridization Oven 640) and rotated at 60 rpm. After hybridization, the microarrays were washed and then stained with the Fluidics Station 450, which was followed by scanning with the Affymetrix GeneChip Scanner 3000 7G (Thermo Fisher Scientific, Waltham, MA, USA), according to the manufacturer’s instructions. The Affymetrix MTA1 analysis was conducted by CapitalBio Technology (Beijing, China). The data were saved and the fluorescence data of the scanned images of the GeneChip were saved as .DAT files for analysis using AGCC software (Affymetrix^®^ GeneChip^®^ Command Console^®^ Software. Affymetrix, Inc. Santa Clara, CA, USA).

#### 4.6.2. Differential Expression Analysis

Differential expression analysis was performed using Transcriptome Analysis Console software 3.0 (Life Technologies | Carlsbad, CA 92008 USA | Toll Free in USA 1 800 955 6288, Thermo Fisher Scientific Inc.). Differentially expressed genes were identified by one-way ANOVA analysis with a *p*-value <0.05 and a fold change of >2 or <0.5.

#### 4.6.3. Quantitative Pathway Analysis

Step 1: Establishment of pathway—differentially expressed gene profile

**Figure 6:**
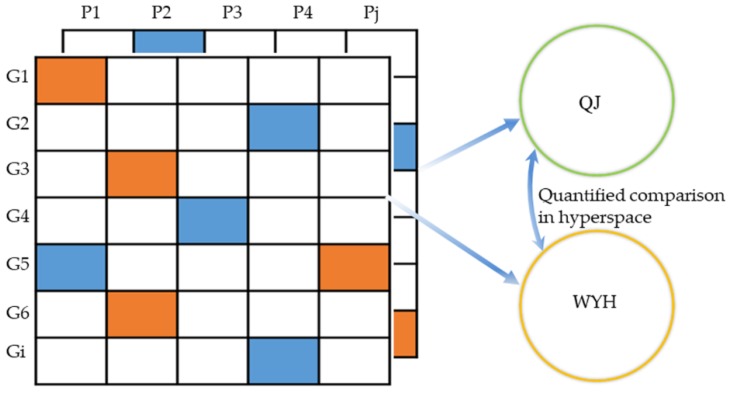
Schematic of the quantitative pathway profile. For the rows *Gi* corresponds to gene *i*. For the columns, *Pj* refers to pathway *j*. The red color means that foldchange >2; the blue color means foldchange <0.5; the white color means genes which are not in pathways.

A two-dimensional table was created with the differentially expressed genes between the drug treatment group and Model group presented as the row vector (Figure 6). The pathways were compared to the column vector. The KEGGREST (R package, based on KEGGSOAP by J. Zhang, R. Gentleman, and Marc Carlson, and KEGG (python package) by Aurelien Mazurie) method was used to determine whether a particular gene belongs to a pathway. If the gene is a member of the pathway, a value was assigned, which is the fold change of the gene to the corresponding position in the table. Otherwise, a value of one was assigned to the corresponding position in the table. In this way, pathways were quantified into digital vectors, which were convenient for comparison and retained the information of gene expression in the pathways (Figure 6).

Step 2: Comparison of pathways between groups in the same dimensional space

After establishing multiple two-dimensional tables of different treatment groups, multiple co-inertia analysis (MCIA) strategies were used for mapping the same pathway of different treatment groups to the same hyperspace. The MCIA can make simultaneous adjustments to pathways and genes of multiple datasets in the same hyperspace [37]. Pathways in different treatment groups that share similar trends are closely projected. According to the relative position of the same pathway in the same hyperspace, the similarities and differences in the pathway between different treatment groups were determined. If the relative position in hyperspace is close, it represents that the pathway was similar between groups, while pathways far away from each other in space have large differences, reflected by the number and expression level of their containing genes. In this way, differences of pathways in different groups were evaluated quantitatively and the mechanism of the formula was decomposed into separate recipes.

### 4.7. Real-Time Fluorescence Quantitative PCR for Verifying the Results of the Trancriptome

The primers used for fluorescent quantitative PCR are shown in the Supplementary Materials in Table S1. TRIzol Reagent (Ambion, 15596026, made in the USA) was used to extract the total RNA. The Revert Aid First Strand cDNA Synthesis Kit (Thermo Fisher Scientific, Waltham, WA, USA,) was used to reverse transcribe the cDNAs. The SYBR Select Master MIX (K1622, Applied Biosystems, 4472908, made in Foster City, CA, USA) was used to determine the expression of the target genes using fluorescent quantitative PCR. The 2^−ΔΔCT^ method was used to analyze gene expression.

### 4.8. Measurement of Indicators by Western Blot

The heart tissue was homogenized in the RIPA LYSIS buffer (50 mM Tris-HCl, pH 7.4, 150 mM NaCl, 2 mM EDTA, 1% NP-40, and 0.1% SDS). Total protein was extracted from this homogenate. The protein concentration in each extract was measured using a protein quantitation kit (ab102535, Abcam, Cambridge, UK; Bradford, Baar, Switzerland) and all samples were adjusted to the same volume with 2 × 4% sodium dodecyl sulfate (SDS) sample buffer. The samples were boiled for 5 min, loaded on a 10% sodium dodecyl sulphate-polyacrylamide gel (50 mg protein/10 mL per well), and separated by electrophoresis for 1.5 h at 80 V and 1.5 h at 120 V using a Bio-Rad mini gel apparatus. The fractionated proteins were transferred to a PVDF membrane using electrophoresis for 1.5 h at 300 mA. The membrane was probed with a primary antibody against Nnt and Nt5e (anti-Nnt antibody, ab110352, Abcam, 1:1000, anti-Nt5e antibody, ab175396, Abcam, 1:1000) and secondary antibody (goat polyclonal secondary antibody against rabbit IgG, #7074s, Cell Signaling Technology, Danvers, MA, USA, 1:1000, horse polyclonal secondary antibody against mouse IgG, #7074s, Cell Signaling Technology, 1:1000). The membrane was then treated with an enhanced chemiluminescence (ECL) reagent (ECL plus Western blotting detection reagent, GE Healthcare, Chicago, IL, USA) for 1 min at room temperature. The bands in the membrane were visualized and analyzed using a UVP Bio Imaging System. After obtaining the density of the GAPDH band, GAPDH (anti-GAPDH antibody, #2118, Cell Signaling Technology, 1:1000) proteins were detected using the same procedure as Nt5e. The final reported data for the Nnt and Nt5e band densities were normalized to GAPDH. The results were analyzed using ImageJ software. Five animal samples were included in each group.

### 4.9. Statistical Analysis

Statistical analyses were performed with the SPSS program (SPSS version 17.0). All data were presented as the mean ± standard error of the mean (SE). Statistical analyses were performed on three or more groups using one-way analysis of variance (ANOVA). The value of *p* < 0.05 was considered statistically significant.

## 5. Conclusions

Quantitative pathway analysis is a novel way for analyzing the differences of functional pathways between recipes of Chinese medicine. Quantitative pathway analysis provides new insights into exploring the molecular mechanisms of QSG treatment on heart failure by decomposing QSG into separate ingredients. QJ and WYH have complementary effects in QSG and play important roles in the myocardial energy metabolism and inflammatory response, respectively. These processes exert therapeutic effects for heart failure.

## Figures and Tables

**Table 1:** Changes in cardiac ultrasound parameters after treatment.

Group	N	LVIDd (mm)	LVIDs (mm)	LVPWd (mm)	LVPWs (mm)	LVAWd (mm)	LVAWs (mm)	LVEDV (μL)	LVESV (μL)	LVM (mg)	EF (%)	FS (%)
Sham	7	4.28 ± 0.09 **	3.12 ± 0.08 **	0.54 ± 0.03 **	0.54 ± 0.02 **	0.51 ± 0.03 **	0.51 ± 0.02 **	82.59 ± 4.02 **	38.82 ± 2.43 **	77.23 ± 6.34 **	52.59 ± 3.02 **	26.98 ± 2.00 **
Model	10	4.85 ± 0.13 ^▲▲^	4.14 ± 0.18 ^▲▲^	0.76 ± 0.02 ^▲▲^	0.73 ± 0.02 ^▲▲^	0.67 ± 0.02 ^▲▲^	0.7 ± 0.02 ^▲▲^	111.41 ± 6.77 ^▲▲^	77.95 ± 8.03 ^▲▲^	142.83 ± 9.43 ^▲▲^	31.51 ± 3.29 ^▲▲^	15.05 ± 1.69 ^▲▲^
QSG	8	4.57 ± 0.10	3.63 ± 0.13 *	0.52 ± 0.01 **	0.51 ± 0.02 **	0.48 ± 0.01 **	0.49 ± 0.02 **	96.11 ± 4.67	56.43 ± 4.69 *	82.00 ± 4.04 **	41.73 ± 2.95 *	20.51 ± 1.62 *
WYH	8	4.53 ± 0.15 *	3.47 ± 0.15 **	0.57 ± 0.15 **	0.63 ± 0.05 *	0.57 ± 0.16 **^,#^	0.56 ± 0.02 **^,#^	94.96 ± 7.36 *	51.01 ± 5.18 **	94.27 ± 6.35 **	46.67 ± 2.78 **	23.39 ± 1.68 **
QJ	8	4.39 ± 0.12 *	3.42 ± 0.11 **	0.54 ± 0.02 **	0.57 ± 0.02 **	0.58 ± 0.02 *^,##^	0.57 ± 0.03 **^,#^	88.24 ± 5.65 *	48.89 ± 3.92 **	88.62 ± 5.82 **	44.66 ± 2.52 **	22.12 ± 1.49 *
Fosinopril sodium	8	4.36 ± 0.17 *	3.32 ± 0.23 **	0.62 ± 0.02 **	0.61 ± 0.02 *	0.57 ± 0.03 **	0.62 ± 0.02 *	86.30 ± 2.80 *	45.11 ± 2.62 **	93.77 ± 4.61 **	47.92 ± 1.85 **	22.99 ± 1.12 **

^▲▲^*p* < 0.01, versus Sham group; * *p* < 0.05, ** *p* < 0.01, versus Model group, ^#^ *p* < 0.05, ^##^ *p* < 0.01, versus QSG group. Data are shown as the mean ± SE. LVIDd: left ventricular internal diameter end diastole; LVIDs: left ventricular internal diameter end systole; LVPWd: left ventricular posterior wall thickness end diastole; LVPWs: left ventricular posterior wall thickness end systole; LVAWd: left ventricular anterior wall thickness end diastole; LVAWs: left ventricular anterior wall thickness end systole; LVEDV: left ventricular end-diastolic volume; LVESV: left ventricular end-systolic volume; LVM: left ventricular mass; EF: ejection fraction; FS: left ventricular fraction shortening.

**Table 2:** The differentially expressed genes of the decomposed recipes (QJ and WYH) in the heart failure-related pathways.

Entrez ID	Gene Symbol	QJ Mean Expression	Fold Change	*p*-Value	WYH Mean Expression	Fold Change	*p*-Value
16000	Igf1	64.23	0.490	0.013↓*	77.62	0.595	0.057↓
108097	Prkab2	467.22	0.457	0.040↓*	515.14	0.459	0.045↓*
21809	Tgfb3	468.04	0.237	0.021↓*	658.34	0.327	0.078↓
21813	Tgfbr2	569.39	0.621	0.515↓	351.72	0.467	0.013↓*
21808	Tgfb2	71.23	0.128	0.012↓*	93.69	0.166	0.019↓*
18115	Nnt	45,948.78	2.120	0.001↑*	37,695.71	1.660	0.008↑
23959	Nt5e	263.38	1.640	0.030↑	313.56	2.040	0.011↑*

The *p*-value of differentially expressed genes are between the treatment group and the Model group. The arrows define the up or down regulation of the gene’s expression. * indicates the gene was significant differentially expressed (Fold change > 2 or Fold change < 0.5. *p*-value < 0.05).

## Supplementary Materials

The following are available online at http://www.mdpi.com/1420-3049/23/7/1829/s1, Table S1: The primers used for fluorescent quantitative PCR (supplied by Shanghai Shenggong Bioengineering Corporation). Table S2: Differentially expressed genes between QSG treatment and Model groups. Table S3: Differentially expressed genes between QJ treatment and Model groups. Table S4: Differentially expressed genes between WYH treatment and Model groups. Table S5: Pathways enriched by differentially expressed genes between QSG treatment and Model groups. Table S6: Pathways enriched by differentially expressed genes between QJ treatment and Model groups. Table S7: Pathways enriched by differentially expressed genes between WYH treatment and Model groups.
